# The Foreign Journals: Midwifery

**Published:** 1841-07

**Authors:** 


### MIDWIFERY.

### *Hemorrhage after Delivery arrested by a new Method.* By Dr. HECKING, of Coblenz.

A WEAK and delicately made woman, soon after she had been quickly and easily delivered of twins, began to have more than usual hemorrhage from the uterus. Permanent pressure on the abdomen, with friction over the uterus, and repeated dashing with cold water were employed, but they were all ineffectual, and the hemorrhage grew more rapid. The author therefore introduced his hand into the uterus (after having dipped it in cold water), and doubling his fist endeavoured, first by rubbing the parietes, and then by compressing them against his other hand, which was placed on the walls of the abdomen to bring on contraction of the uterus. But this plan also was altogether useless; the uterus, still remaining distended, was every instant filled with fluid and coagulated blood. After all attempts had proved vain, and when the patient's danger was now extreme, the author took a sponge dipped in cold water, and then squeezed it on the internal surface of the uterus, so as completely to wet it all over, at the same time maintaining the external pressure. As often as the sponge became covered with coagulated blood, it was taken away, washed with cold water, and again introduced as quickly as possible. By this means several times repeated (for in the powerless condition of the patient it was not a difficult thing to do,) the hemorrhage was at last arrested, and the woman's life was saved.

*Casper's Wochenschrift. November* 28, 1840.

---

### *Hernia of the Uterus, and Cæsarean Operation: Mother and Child saved.* By M. LEDESMA, of Salamanca.

ON the 26th of January, 1839, a female named Ramus, 24 years of age, of good constitution, having had six children, stated that some time before her marriage she had an inguinal enterocele on the right side. When the seventh pregnancy had advanced to the third month, she suddenly perceived an unpleasant dragging sensation at the bottom of the left side of the abdomen; the abdominal tumour disappeared, and she voided some blood by the vagina. Something unusual, hard, and painful on pressure was now discovered in the site of the inguinal hernia. She frequently attempted reduction in vain. Seven weeks afterwards she perceived evident movements. The tumour descended from the bottom and right side of the abdomen on the thigh of that side, distending the right labium pudendi, falling on the pubis and crossing towards the left thigh; it was 22 inches in extent, 25 in circumference at its middle, and 22½ at its junction with the abdomen. It was fluctuating, and on displacing the liquid, a hard body was perceived which appeared moveable in the liquid. The neck of the uterus could not be found in the vagina. At seven months the sounds of the fœtal heart, and the placentar souffle when examined by the stethoscope were perfectly distinct from the superficial position of the uterus. M. Ledesma judged by the souffle that the placenta was inserted to the left and a little anteriorly: the sound was clear and very circumscribed. Attempts at reduction were useless. On the 6th July she experienced the first pains. On the 7th the membranes broke, and the waters were discharged by the vagina. Reduction again attempted in vain. Hysterotomy was practised, with care to avoid the left lateral part of the tumour. A great quantity of blood followed the incision of the uterus. The head of the fœtus was against the cervix, the surgeon seized the feet which were at the fundus and forcibly withdrew the child. The placenta was at the place foreseen. After the uterus was emptied a tepid bath was prescribed, which excited uterine contractions and arrested all hemorrhage. Reduction of the uterus was thought to be impossible, and it was left externally.

July 12. The lochia take the route of the vagina. After some accidents, slight in comparison with the severity of the operation, the patient's health is reestablished. The wound suppurated more than a month.

July 13. The catamenia reappeared.

August 11. Convalescence is complete. The tumour has considerably diminished. The infant was well, but died some time afterwards.

*Gazette Médicale de Paris, 7 Novembre,* 1840.

(*Taken from the Journal de la Société de Médecine Pratique de Montpellier.*)

---

*Retroversion of the Unimpregnated Uterus.* By Dr. Alken, of Bergheim.

A young woman, twenty-six years of age, accustomed to hard work, found herself suffering one day from difficulty in passing her urine and fæces; and these gradually increased till, after fourteen days, there was complete retention of urine and of fæces. When the author was called in he found the patient with a pale fallen countenance, cold extremities, a small, rapid, jarring pulse, hurried respiration, insatiable thirst for cold water, hiccup, vomiting, &c. The abdomen was very tense, the urinary bladder distended up to the umbilicus, and every movement of the abdomen and the slightest touch extremely painful. Examination detected a complete retroversion of the uterus, so that its vaginal portion was immoveably fixed against the pubes, and its fundus was thrust deep into the pelvis. The patient had been in this state for ten hours. The urine was with difficulty drawn off by the catheter, and after bleeding and the warm bath, an attempt was made to reduce the uterus to its right position by pressing it in opposite directions through the medium of the vagina and the rectum. After the efforts had been continued an hour the uterus returned to its place. The replacement was perfect; but on the following day the retroversion again occurred after some exertion. It was again reduced with much greater facility than before, and by observing the horizontal posture for nearly three weeks, it was prevented from again returning. The patient was watched for several months, and it was clearly determined that at the time of the retroversion the uterus was in the unimpregnated state.

*Casper's Wochenschrift. April* 3, 1841.

---

*Extraction of a Foreign Body implanted in the Uterus.* By M. Maisonneuve, of the Hospital St. Louis.

This patient was thirty years of age on her admission to the hospital, Sept. 14, 1840. At the age of twenty-eight she was in good health, became pregnant, and in the fifth month of gestation states that she miscarried and suffered from severe metro peritonitis. She was obliged to enter the hospital La Pitié soon after she had begun to leave her room, where the surgeon diagnosticated metritis with hypertrophy of the anterior surface of the uterus. Various means were persevered in without effect, and when she came under the care of M. Maisonneuve her general powers were enfeebled, digestion bad, hectic at night, and she had dull continued pains in the loins and hypogastrium, which latter region was occupied by an irregular hard tumour, slightly painful on pressure, which filled the pelvis and extended into the iliac fossa. The os uteri permitted the entrance of the finger, but the body and neck of the organ were lost in an irregular, hard, and absolutely immoveable mass. Examined by the speculum the vagina appeared to be in the normal state, and the only unusual appearance was abnormal patency of the os uteri, which permitted the surgeon to see something whitish. He passed a stylet to discover the nature of this substance, and was astonished to find that he could circumscribe the unknown object by passing the stylet before and behind it. It adhered to the lips of the os uteri on all sides.

Persuaded that this was a foreign body implanted in the walls of the uterus,

M. Maisonneuve first endeavoured to divide it with scissors but could not. He then placed one beak of a pair of long polypus forceps behind and the other before it, and by careful traction removed, without causing much pain, a piece of wooden stick, 122 millimeters in length, pointed at one extremity and bent at the other. Looking to her account of the case, it appeared highly probable that this stick had been broken in the uterus during criminal efforts to produce abortion, and this opinion has been since confirmed.

The operation was followed by a return of abdominal and lumbar pains, and great febrile reaction, which disappeared in about eight days under general bleeding, baths, and several applications of leeches. But there remained a tumour in the pelvis which probably resulted from chronic adhesions of the uterus, bladder, and rectum, with some of the intestines. However by the 1st of January, 1841, the tumour had greatly diminished and the general health was much improved.

*Gazette Médicale de Paris. Avril* 3, 1841.

---

*Experiments on Uterine Injections.* By M. HOURMANN.

1*st series.* Injections of the uterus separated from the body. Eleven experiments were made. Five times the liquid traversed the fallopian tubes and was effused into the peritoneum; in the other six the tubes were not distended, or but slightly.

2*d series.* Injections of the uterus *in situ.* In five experiments, the liquid passed through the tubes.

3*d series.* Injections of the uterus *in situ* some days after delivery. Injections passed with force from large syringes never penetrated the tubes. The mucous membrane of the uterus and tubes was generally tumefied and red. Four of these experiments were made by M. Danyau.

Among the six cases of non-penetration in the first series, three were in females lately delivered. These seven cases, then, show that uterine injections may be employed without danger after accouchement.

*Gazette Médicale de Paris. Février* 20, 1841.

---

*On the Microscopic Characters of healthy Milk.* By Pr. Von D'OUTREPONT, of Würzburg.

HE has recently put to the test some of M. Donné's statements with reference to the characteristics of healthy milk, and has arrived at somewhat different conclusions, though he fully confirms Donné's statements with reference to the difference of the corpuscles in the colostrum and milk.

Professor Von D'Outrepont found that in the greater number of instances the peculiar granular bodies of the colostrum (*corps granuleux*) disappeared on the third day after delivery and not on the sixth or tenth as stated by Donné. Even in those cases, however, in which they could still be detected on the tenth or twelfth day, the milk produced no injurious effects on the infant; nor did it indeed in some instances in which the milk retained the characters of colostrum so long as a month after delivery. The milk of a female labouring under severe metro-peritonitis presented the characters of true milk, not of colostrum. That likewise from the left breast of a person whose right breast was in a state of suppuration presented all the characters of healthy milk, though pus-globules were mixed with the milk in the other breast. In two instances where the breasts became inflamed without suppurating, the milk continued to present all characters of the healthy secretion and did not contain any of the granular bodies of the colostrum.

Professor D'Outrepont had the opportunity of examining the milk of a woman who, after having suckled her third child for some months, began to menstruate regularly. During the flow of the menses the child became indisposed to suck and suffered from vomiting, but recovered its health immediately on their cessation. During menstruation the milk possessed all the characters of colostrum, while at other times its appearance was precisely that of healthy milk. The secretion from the breasts of a woman who had never been pregnant presented all the peculiarities of colostrum; that contained in the breasts of another woman who never suckled her children, though the glands were always full except during pregnancy, differed in no respect from healthy milk.

*Neue Zeitschrift für Gebertskunde. Bd.* x. *Heft* i.

